# Perceived decent work and work engagement among nurses: a multicenter cross-sectional mediation analysis of self-esteem

**DOI:** 10.3389/fpubh.2026.1947395

**Published:** 2026-09-07

**Authors:** Jiayu Liu, Tao Lin, Tongxin Zhang, Bei Yang, Qin Zeng

**Affiliations:** 1 Department of Pediatric Gastroenterology Nursing, West China Second University Hospital, Sichuan University, Chengdu, Sichuan, China; 2 Key Laboratory of Birth Defects and Related Diseases of Women and Children, Sichuan University, Ministry of Education, Chengdu, Sichuan, China; 3 Emergency Department of West China Hospital, Sichuan University, Chengdu, China; 4 West China School of Nursing, Sichuan University, Chengdu, China

**Keywords:** decent work, health workforce policy, health-system resilience, nurses, occupational health, self-esteem, work engagement

## Abstract

**Background:**

A stable and engaged nursing workforce is important for continuity of care and health-system preparedness. Perceived decent work may be associated with nurses’ work engagement, and self-esteem may account statistically for part of this association; however, cross-sectional data cannot establish temporal or causal pathways.

**Objective:**

To assess perceived decent work among nurses in tertiary Grade-A hospitals in Sichuan Province, examine its association with work engagement, and estimate the statistical indirect association through self-esteem.

**Methods:**

We conducted a multicenter cross-sectional survey between November 2022 and May 2023 among 323 registered clinical nurses recruited by convenience sampling from five hospitals with different administrative affiliations in Sichuan Province. Spearman correlations described unadjusted monotonic associations. Multiple linear regression estimated adjusted associations after simultaneous control for gender, age, household registration, education, and professional title. A bias-corrected bootstrap mediation analysis with 5,000 resamples estimated total, direct, and indirect associations; a sensitivity model adjusted for gender, age, education, and professional title.

**Results:**

Mean scores for perceived decent work, self-esteem, and work engagement were 39.27 ± 7.62, 32.52 ± 5.00, and 27.27 ± 6.53, respectively. The variables were positively correlated (*r* = 0.336–0.637, all *p* < 0.01). The total, direct, and indirect estimates were 0.637 (95% CI 0.552–0.721), 0.566 (95% CI 0.468–0.664), and 0.071 (95% CI 0.029–0.121), respectively; the indirect estimate represented 11.1% of the total and remained statistically significant after covariate adjustment (0.065, 95% CI 0.024–0.115). In adjusted regressions, male nurses had lower perceived decent work (*B* = −3.447, 95% CI −5.606 to −1.288), self-esteem (*B* = −2.020, 95% CI −3.446 to −0.594), and work engagement (*B* = −2.656, 95% CI −4.524 to −0.789) scores than female nurses.

**Conclusion:**

In this regional convenience sample, perceived decent work was positively associated with work engagement, with a modest statistical indirect association through self-esteem. The cross-sectional design precludes conclusions about temporal ordering, causality, intervention effects, or population-level policy effectiveness. The findings support prospective evaluation of organizational and workforce hypotheses in more representative samples.

## 1 Introduction

As the largest occupational group within the health workforce, nurses play an indispensable role in delivering universal health coverage, ensuring equitable access to essential services, and maintaining continuity of care during public health emergencies. According to the World Health Organization’s State of the World’s Nursing 2025, investing in nursing education, employment, leadership, service delivery, and working conditions should be treated as a policy priority. Meanwhile, the Working for Health 2022–2030 Action Plan connects a well-supported health and care workforce with stronger health-system resilience, improved emergency preparedness, and better population health outcomes (1, 2). Consequently, nursing workforce stability is not merely an internal hospital management concern: when disengagement and attrition persist, they can erode surge capacity, diminish service availability, and compromise public health systems’ ability to address both routine needs and crisis-related demands.

Despite their pivotal role in healthcare, nurses often grapple with overwhelming workloads, unbalanced nurse-to-patient ratios, limited career growth prospects, inadequate recognition, and even workplace violence (3–9). These challenges mirror the International Labor Organization’s decent-work agenda for nurses, which advocates for fair pay, safe working conditions, reasonable hours, social protection, worker participation, and respect for rights (10). From a health workforce policy standpoint, ensuring decent work is not just a labor standard but also a key factor influencing nurses’ psychological commitment and ongoing engagement with their profession.

The notion of perceived decent work reflects workers’ personal evaluations of whether their jobs provide sufficient rewards, respect, career growth opportunities, recognition, a supportive workplace, and reasonable working hours (11–13). In nursing, this evaluation is influenced by daily organizational practices, as well as larger regulatory and labor market factors. Studies indicate that when nurses perceive their work as decent, they tend to be more satisfied with their jobs, identify more strongly with their profession, and are more likely to stay in it (5–9). Work engagement, characterized by energy, commitment, and focus, is especially important in this context, as it drives nurses to provide consistent, high-quality care (14). While maintaining engagement can enhance patient safety, service reliability, and organizational preparedness, conclusive evidence linking these outcomes directly to engagement is still lacking.

Self-esteem may offer a useful framework for understanding how employment conditions translate into work engagement. Self-Determination Theory posits that autonomy, competence, and relatedness together support intrinsic motivation (15), while self-concept perspectives emphasize that recognition and respectful treatment shape how individuals evaluate their own worth (16). When nurses perceive that they are treated fairly, respected professionally, and provided with credible opportunities for development, they may develop a stronger sense of self-worth and professional confidence. These psychological resources may, in turn, help them persevere and remain dedicated even under demanding clinical conditions (17, 18). Conversely, prolonged exposure to inequitable or unsafe working conditions may undermine both self-evaluation and engagement.

Previous studies have examined how nurses perceive decent work in psychiatric, emergency, pediatric, and tertiary hospitals (5–8, 19), noting that professional mission, organizational self-esteem, and professional respect may link employment conditions to job outcomes (8, 18). However, the role of self-esteem in mediating the link between decent work perception and work engagement is not well-explored, especially beyond single hospitals. Multi-center research could reveal if this relationship holds across diverse hospitals and pinpoint which staff groups need focused policy support.

Accordingly, the present study surveyed nurses across five tertiary Grade-A hospitals in Sichuan Province, China, to assess perceived decent work, examine its association with work engagement, and estimate whether self-esteem statistically accounts for part of that association. We hypothesized that perceived decent work would be positively associated with work engagement (H1) and self-esteem (H2), that self-esteem would be positively associated with work engagement (H3), and that the cross-sectional model would yield a nonzero statistical indirect estimate through self-esteem (H4). The findings are intended to inform nursing-management hypotheses and candidate workforce indicators for future prospective evaluation, rather than to demonstrate causal mechanisms or policy effectiveness.

## 2 Materials and methods

### 2.1 Study design

Between November 2022 and May 2023, a multicenter cross-sectional survey was conducted in five tertiary Grade-A hospitals in Sichuan Province, China: two hospitals directly under the National Health Commission, one provincial hospital, one municipal hospital, and one district/county hospital. This affiliation mix increased institutional variation within the study frame but did not create a probability sample or establish representativeness of hospitals or nurses in Sichuan Province or China. The study received ethical approval from the Biomedical Ethics Review Committee of West China Hospital, Sichuan University [Approval No. 2021 Review (966)] and adhered to the Declaration of Helsinki. Before participation, all respondents were informed of the study objectives, procedures, anonymity, confidentiality, voluntary participation, and right to withdraw. Written informed consent was obtained before questionnaire completion.

### 2.2 Participant recruitment and eligibility criteria

The sampling frame comprised five tertiary Grade-A hospitals selected to cover different administrative affiliations in Sichuan Province. Within this multicenter frame, eligible clinical nurses were recruited by convenience sampling through local nurse representatives. Neither hospitals nor individual nurses were selected through probability sampling, and non-tertiary hospitals and institutions outside Sichuan Province were not included.

The inclusion criteria were as follows: (1) currently active registered nurses; (2) having at least 6 months of clinical work experience; (3) providing informed consent and voluntarily agreeing to participate in this study.

The exclusion criteria were: (1) individuals on maternity leave, sick leave, or personal leave during the survey period; (2) those who had submitted resignation applications but had not yet officially left the hospital; (3) those who failed to complete the questionnaire in full.

Additionally, to safeguard data quality, questionnaires completed in less than 60 s were excluded during the data-cleaning phase. This threshold was determined based on pre-test results: in the pre-test, all valid questionnaires had completion times exceeding 75 s. Given that the questionnaire in this study contains 31 scale items, 60 s was established as the screening threshold, as it falls below the normal time required to read and respond.

When determining the sample size for cross-sectional questionnaire studies involving multivariate analysis, a common rule of thumb is to use 5–10 times the number of scale items. In this study, the three main scales comprised 31 items in total, yielding a target sample size of 155–310 cases. To account for potential invalid responses and to ensure sufficient data for robust model analysis, the study ultimately distributed 400 questionnaires.

### 2.3 Measurement tools

The questionnaire used in this study comprises a general information questionnaire, the Decent Work Perception Scale, the Self-Esteem Scale, and the Work Engagement Scale.

#### 2.3.1 General information questionnaire

The research team developed the general information questionnaire to collect participants’ demographic and occupational details, specifically gender, age, household registration type, educational level, and professional title.

#### 2.3.2 Decent work perception scale

The Decent Work Perception Scale, developed by Mao et al. (20), was employed to gauge perceived decent work. This scale encompasses five key aspects: work rewards, job position, career development, professional recognition, and work environment. It comprises 12 items. Examples include: “My job makes me proud before family and friends,” “My promotions haven’t matched expectations lately,” and “I’m often overloaded with work.”

This scale uses a 5-point Likert rating system, where each item is scored from 1 to 5, resulting in a total score range of 12–60. Notably, items 6 through 9 are negatively phrased and require reverse-scoring before summing. A higher total reflects a greater perception of decent work among nurses. The scale demonstrates strong internal consistency, evidenced by Cronbach’s alpha values of 0.87 originally, 0.821 in pre-testing, and 0.8701 in the main study.

#### 2.3.3 Self-esteem scale

To gauge self-esteem, we used the Rosenberg Self-Esteem Scale (RSES), originally developed by Rosenberg (16) and later adapted into Chinese by Ji and Yu (21). While the original scale used a 4-point scoring system, we adapted it to a 5-point Likert scale to align with our questionnaire’s standardized format, while keeping the core item content intact. This Chinese iteration is widely used across China and demonstrates strong reliability and validity. Our study reaffirmed its internal consistency. Comprising 10 items, the scale delves into individuals’ holistic perceptions of their worth and capabilities. Sample statements include: “I find few things to be proud of,” “I aspire to garner greater self-respect,” and “I frequently feel worthless.”

This scale uses a 5-point Likert format, where individual items are scored from 1 to 5, yielding a total score range of 10 to 50. Notably, items 20, 22, 25, 26, and 27 are negatively phrased and require reverse coding before calculating the total. A higher score indicates stronger self-esteem among nurses. The scale’s reliability is robust, with Cronbach’s alpha values of 0.88 for the original, 0.808 for the pre-survey, and 0.7275 in the main study.

#### 2.3.4 Work engagement scale

To evaluate work engagement, this study employed the UWES-9 short form, a tool crafted by Schaufeli et al. (14). We used the Chinese adaptation of this scale, based on Li et al.’s work (22). To maintain consistency with the questionnaire’s scoring norms, we modified the original 7-point response scale (0–6) to a 5-point Likert scale (1–5), while keeping the item content and three-dimensional framework intact. The Chinese version of the scale has proven reliable and valid, and our retest on the current study sample further confirmed its dependable internal consistency, making it apt for our research. The scale encompasses three facets: vigor, dedication, and absorption, and comprises nine items. Sample items are: “I approach my job with zeal,” “My work sparks creativity,” and “I take pride in my accomplishments.”

The scale uses a 5-point Likert scale, where each item is rated from 1 to 5, resulting in a total score range of 9–45. All items are positively scored without any reverse-scored items. A higher total score indicates a stronger work engagement among nurses. The scale demonstrates excellent internal consistency, with Cronbach’s alpha values of 0.93, 0.849, and 0.8729 in the initial, pre-survey, and formal study phases, respectively.

### 2.4 Data collection and quality control

#### 2.4.1 Questionnaire design and procedural control

The questionnaire in this study consists of a general information questionnaire together with mature scales from both domestic and international sources. The first section collects demographic and occupation-related information, including gender, age, household registration type, education level, and professional title; the second section is the Decent Work Perception Scale; the third section is the Self-Esteem Scale; and the fourth section is the Work Engagement Scale.

To counter common method bias, we took several steps during questionnaire creation and distribution. We allowed anonymous responses to curb social desirability bias. We incorporated reverse-scored items into the scale to discourage consistent responding. The instructions stressed that there were no correct or incorrect answers, urging participants to answer independently based on real feelings. Moreover, all questionnaire items were mandatory to minimize missing data.

#### 2.4.2 Data collection process

Data were collected using online questionnaires. The researchers sent the QR code or link generated by the Wenjuanxing platform to nurse representatives at each hospital, who helped forward it to nurses who met the inclusion criteria. The first page of the questionnaire provided unified instructions explaining the research purpose, completion requirements, anonymity, confidentiality, and voluntary participation principles. Participants confirmed informed consent before accessing the formal questionnaire and selected the most appropriate option based on their actual situation.

Procedural safeguards were used to reduce information and participation pressures: the instructions stated that the study was solely for academic research, data were anonymous, no personally identifying information was collected, and participation or refusal would not affect work evaluation. These safeguards could not eliminate selection bias arising from voluntary online participation, distribution through nurse representatives, or differential willingness to respond.

#### 2.4.3 Data organization and quality control

After completing data collection, the researchers exported all raw data from the Wenjuanxing platform in Excel format. The exported data included participants’ response results and questionnaire completion times. Data cleaning and quality control mainly involved the following steps: (1) checking whether sample serial numbers were continuous and identifying and deleting duplicate records; (2) checking whether core variables and scale items had missing values; (3) verifying that all 31 scale items fell within the theoretical value range of 1–5 points; (4) identifying questionnaires that might present mechanical response risks based on questionnaire completion time, item standard deviation, highest same-option ratio, and number of options used; (5) reverse-scoring negative items and calculating the total score for each scale.

We sent 400 questionnaires and retained 323 valid responses, achieving an 80.75% response rate. After careful data checks, we sequentially numbered these responses with no repeats. Core variables had no missing data, and all scale items scored between 1 and 5 as expected.

### 2.5 Statistical analysis

Data analysis was performed using Python version 3.12 and SPSS version 30.0. The statistical analysis mainly encompassed reliability testing, structural validity testing, common method bias testing, descriptive statistics, inter-group difference analysis, correlation analysis, multiple linear regression analysis, and mediation effect testing.

Initially, Cronbach’s alpha was used to assess the internal consistency of each scale. The Kaiser–Meyer–Olkin test and Bartlett’s test of sphericity were then used to assess suitability for factor analysis. Harman’s unrotated single-factor test was used as a limited diagnostic for common method bias, with a first-factor explanation rate below 40% interpreted as no dominant single factor; this screen cannot exclude common method bias.

For descriptive statistics, categorical variables were expressed as frequencies and percentages; continuous variables were described using mean, standard deviation, median, and interquartile range, depending on their distribution. For two-group comparisons, an independent-samples *t*-test was used when continuous variables were approximately normally distributed with homogeneous variance; Welch’s *t*-test was used when variance was heterogeneous; and the Mann–Whitney *U* test was used when the data did not conform to a normal distribution. For comparisons involving multiple groups, one-way ANOVA was employed when the data approximated a normal distribution with equal variances; the Kruskal-Wallis *H* test was applied when parametric assumptions were not satisfied. Where multi-group comparisons yielded statistically significant results, Bonferroni adjustments were subsequently implemented for pairwise *post hoc* analyses.

Spearman’s rank correlation was used to examine correlations between perceived decent work, self-esteem, and work engagement. To further evaluate the unique contributions of demographic and job-related factors to the primary outcome measures, multiple linear regression analyses were performed. A two-tailed significance level of *P* < 0.05 was applied throughout.

**Figure 1:**
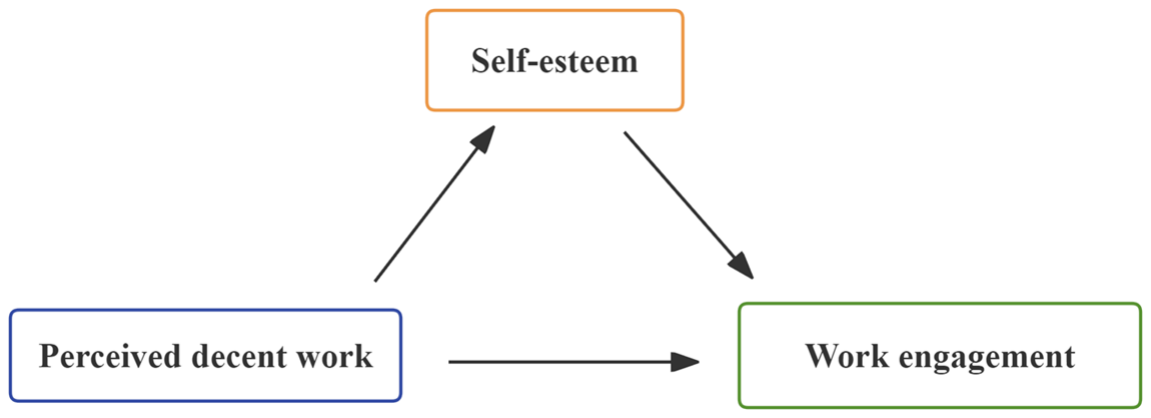
Hypothesized mediation model linking perceived decent work, self-esteem, and work engagement.

Based on the theoretical framework, the statistical mediation model specified perceived decent work as X, self-esteem as M, and work engagement as Y. The model estimated the total association (c), the direct association conditional on self-esteem (c’), and the indirect association (a × b). Bias-corrected bootstrap confidence intervals were calculated from 5,000 resamples, and an indirect estimate was considered statistically different from zero when its 95% confidence interval excluded zero. The primary model was unadjusted; a sensitivity model included gender, age, education, and professional title as covariates. Household registration was included in the multiple regression analyses but not in the reported mediation sensitivity model (Figure 1). The X-M-Y order reflects the study hypothesis only; because all variables were measured at one time point, it does not establish temporal sequence or causal mediation.

## 3 Results

### 3.1 General characteristics of participants

This study included 323 nurses. Participants were predominantly female (264, 81.73%), with 59 male participants (18.27%). The largest age groups were 26–29 years (148, 45.82%) and 20–25 years (111, 34.37%). Rural household registration was reported by 180 participants (55.73%) and urban registration by 143 (44.27%). Most participants held a bachelor’s degree (218, 67.49%). Professional titles included nurse practitioner (129, 39.94%), staff nurse (114, 35.29%), charge nurse or above (49, 15.17%), and standardized training nurse (31, 9.60%) (Table 1). The sample included hospitals across four administrative-affiliation levels; this describes institutional coverage and should not be interpreted as population representativeness.

**Table 1:** General characteristics of participants (*N* = 323).

Characteristic	Category	Frequency	Percentage (%)
Gender	Female	264	81.73
	Male	59	18.27
Age	20–25 years	111	34.37
	26–29 years	148	45.82
	30–35 years	42	13.00
	≥36 years	22	6.81
Household registration	Rural	180	55.73
	Urban	143	44.27
Education level	Junior college	78	24.15
	Bachelor’s degree	218	67.49
	Master’s degree or above	27	8.36
Professional title	Standardized training nurse	31	9.60
	Nurse	114	35.29
	Nurse practitioner	129	39.94
	Charge nurse and above	49	15.17

### 3.2 Reliability, structural applicability, and common method Bias testing

After reverse-scoring correction, Cronbach’s alpha coefficients for the Decent Work Perception Scale, Self-Esteem Scale, and Work Engagement Scale were 0.870, 0.728, and 0.873, respectively. The KMO value was 0.919, and Bartlett’s test of sphericity was statistically significant (chi-square = 3817.448, df = 465, *p* < 0.001). In Harman’s unrotated single-factor analysis, the first factor explained 28.92%, below the prespecified 40% threshold. This result did not indicate a dominant single factor but does not rule out common method bias from collecting all variables by self-report in the same survey.

### 3.3 Descriptive statistics of core variables

The perceived decent work score of 323 nurses was 39.27 ± 7.62 points, the self-esteem score was 32.52 ± 5.00 points, the work engagement score was 27.27 ± 6.53 points, and the total score of the three scales was 99.06 ± 15.41 points. The absolute values of skewness and kurtosis of each variable were relatively small, suggesting that the distribution of core variables showed no obvious skewness (Table 2).

**Table 2:** Descriptive statistics of core variables (*N* = 323).

Variable	*N*	Mean	SD	Min	P25	Median	P75	Max	Skewness	Kurtosis
Perceived decent work	323	39.27	7.62	13	34.50	40	45	57	−0.336	−0.042
Self-esteem	323	32.52	5.00	17	29.50	32	36	46	−0.125	−0.065
Work engagement	323	27.27	6.53	9	23.00	28	32	45	−0.151	−0.328
Total score	323	99.06	15.41	55	88.00	100	111	140	−0.262	−0.464

### 3.4 Comparison of core variable scores among nurses with different characteristics

Inter-group difference analysis revealed statistically significant differences in perceived decent work, self-esteem, work engagement, and the total score of the three scales among nurses of different genders. Differences in perceived decent work and work engagement among nurses of different ages were statistically significant; differences in perceived decent work among nurses of different education levels were statistically significant; and differences in perceived decent work, self-esteem, and work engagement among nurses of different professional titles were statistically significant. The differences in each core variable among nurses of different household registration types were not statistically significant.

Bonferroni-corrected *post hoc* comparisons showed that nurses aged 20–25 years had lower perceived decent work and work engagement scores than those aged 26–29 years (adjusted *p* = 0.0392 and 0.0415, respectively). Nurses in the staff-nurse group had lower perceived decent work scores than nurse practitioners (adjusted *p* = 0.0089) (Table 3).

**Table 3:** *Post hoc* comparisons remaining significant after Bonferroni correction.

Grouping variable	Outcome variable	Group 1	Group 2	Adjusted *p*-value	Direction of mean difference
Age	Perceived decent work	20–25 years	26–29 years	0.0392	Lower in 20–25 years group
	Work engagement	20–25 years	26–29 years	0.0415	Lower in 20–25 years group
Professional title	Perceived decent work	Nurse	Nurse practitioner	0.0089	Lower in Nurse group

### 3.5 Correlation analysis and multiple linear regression analysis

**Figure 2:**
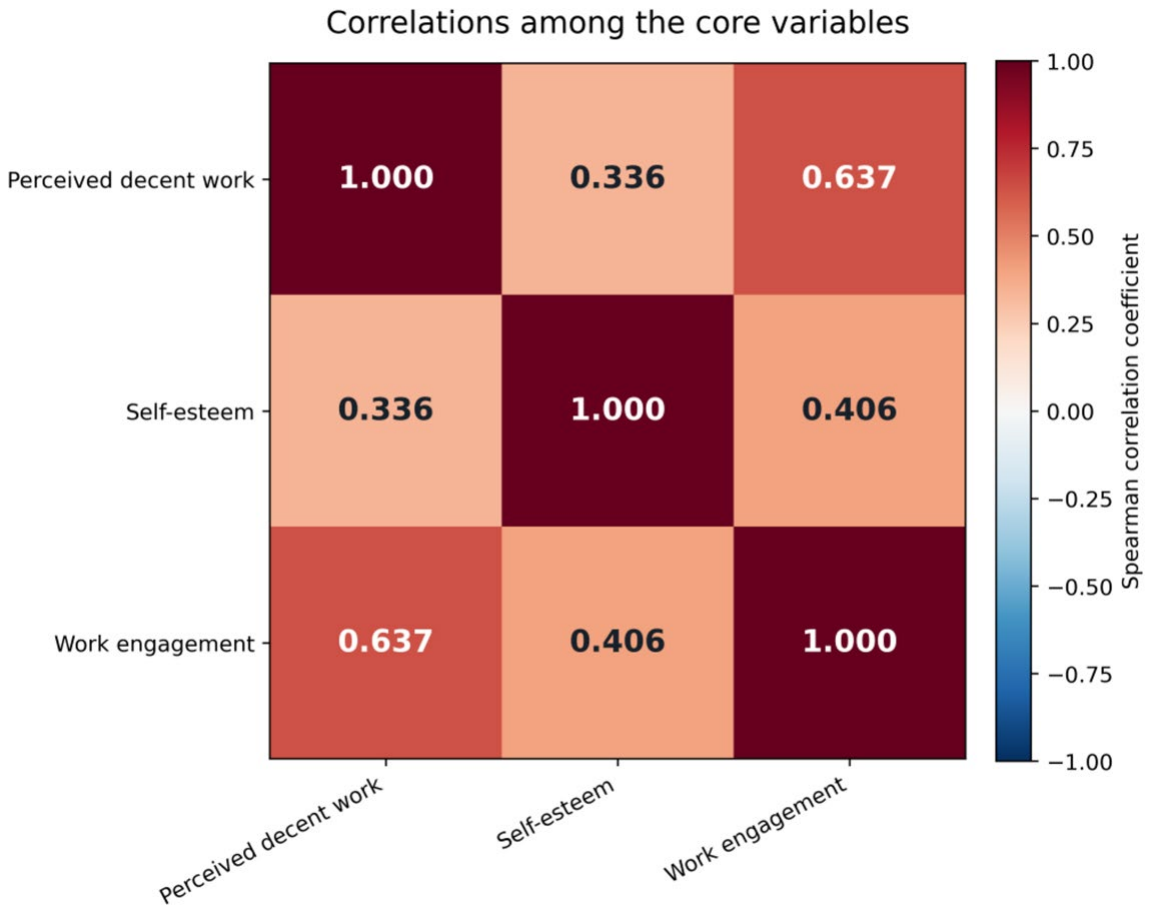
Spearman correlations among perceived decent work, self-esteem, and work engagement. All off-diagonal correlations were statistically significant at *p* < 0.01.

Spearman correlation analysis showed positive monotonic associations among perceived decent work, self-esteem, and work engagement. Perceived decent work was correlated with work engagement (*r* = 0.637), self-esteem was correlated with work engagement (*r* = 0.406), and perceived decent work was correlated with self-esteem (*r* = 0.336) (Figure 2). These unadjusted coefficients describe association strength and do not indicate temporal ordering or causality.

With gender, age, household registration, education, and professional title entered simultaneously, male sex was associated with lower scores than female sex for perceived decent work (*B* = −3.447, SE = 1.097, 95% CI −5.606 to −1.288), self-esteem (*B* = −2.020, SE = 0.725, 95% CI −3.446 to −0.594), work engagement (*B* = −2.656, SE = 0.949, 95% CI −4.524 to −0.789), and the combined three-scale score (*B* = −8.123, SE = 2.239, 95% CI −12.529 to −3.717) (Table 4). These coefficients are adjusted mean-score differences within this sample.

**Table 4:** Multiple linear regression analysis of core variables.

Outcome	Predictor	*B*	SE	*t*	*p*	95% CI low	95% CI high	*R* ^2^	Adjusted *R*^2^
Perceived decent work	Gender (male)	−3.447	1.097	−3.141	0.0018	−5.606	−1.288	0.0857	0.0534
Self-esteem	Gender (male)	−2.020	0.7248	−2.787	0.0057	−3.446	−0.5937	0.0733	0.0406
Work engagement	Gender (male)	−2.656	0.9492	−2.799	0.0054	−4.524	−0.7890	0.0686	0.0357
Total score of three scales	Gender (male)	−8.123	2.239	−3.628	0.0003	−12.529	−3.717	0.0701	0.0372

Gender, age, household registration, education, and professional title were included as independent variables. Only statistically significant variables are shown; female nurses were the reference group.

With gender, age, household registration, education, and professional title entered simultaneously, no age category was significantly associated with perceived decent work, self-esteem, work engagement, or the combined three-scale score (all *p* > 0.19), with 26–29 years as the reference group.

### 3.6 Mediation effect testing

The bias-corrected bootstrap model (5,000 resamples) estimated a total association of 0.637 (95% CI 0.552–0.721), a direct association conditional on self-esteem of 0.566 (95% CI 0.468–0.664), and an indirect association of 0.071 (95% CI 0.029–0.121). The indirect estimate represented 11.1% of the total estimate (Table 5). The confidence interval excluded zero, supporting a nonzero statistical indirect association in this sample. This decomposition is descriptive and should not be interpreted as evidence that changes in perceived decent work caused changes in self-esteem or work engagement.

**Table 5:** Mediation effect test results.

Path	Effect value	Standard error	95% CI (Lower, Upper)	Mediation proportion (%)
Total effect (c)	0.637	0.043	(0.552, 0.721)	—
Direct effect (c’)	0.566	0.050	(0.468, 0.664)	88.9%
Indirect effect (a × b)	0.071	0.023	(0.029, 0.121)	11.1%

**Figure 3:**
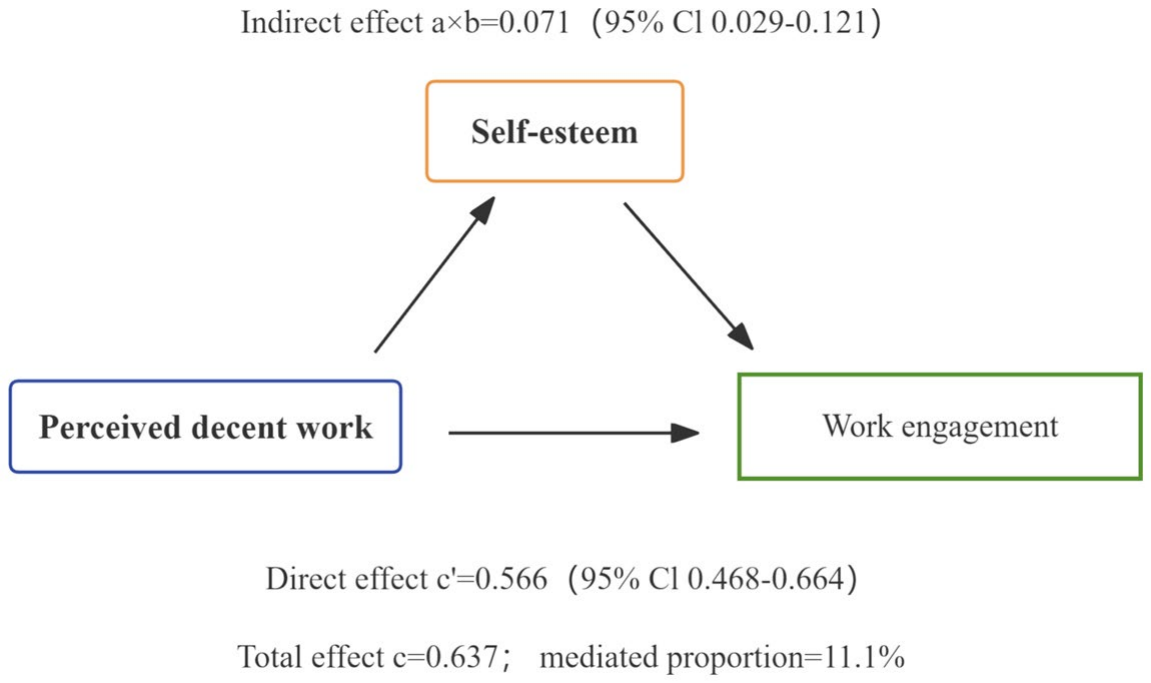
Mediation analysis for the association between perceived decent work and work engagement through self-esteem. The indirect effect remained significant after adjustment for gender, age, education, and professional title (0.065, 95% CI 0.024–0.115).

In the sensitivity model adjusted for gender, age, education, and professional title, the indirect estimate was 0.065 (95% CI 0.024–0.115). Its confidence interval also excluded zero. The similarity of the adjusted and unadjusted indirect estimates indicates stability to this measured covariate set, but it does not address unmeasured confounding, reverse directionality, or the absence of temporal ordering (Figure 3).

## 4 Discussion

### 4.1 Analysis of the current status of nurses’ perceived decent work, self-esteem, and work engagement

The total perceived decent work score among nurses in the five participating tertiary Grade-A hospitals was 39.27 ± 7.62, corresponding to a mean item score of approximately 3.27 and suggesting a moderately low level within this sample. This finding is consistent with reports by Tang et al. (5), Zhao (23), and Tong et al. (24), and identifies scope for examining nurses’ appraisal of professional worth, dignity, career opportunities, and workplace conditions. High workload, imbalanced nurse–patient ratios, limited career pathways, and insufficient recognition are plausible contextual explanations, but they were not tested as causal determinants in this study. Because participants were recruited by convenience from five tertiary hospitals in one province, the results cannot establish that low perceived decent work is universal across hospital types, Sichuan Province, southwest China, or China. It instead provides a regional signal that warrants confirmation in probability-based and geographically broader samples.

In this study, nurses’ self-esteem scale score was 32.52 ± 5.00 points, indicating that the overall sense of self-worth of the research subjects was acceptable. However, there were still certain differences among individuals. Self-esteem represents an individual’s global subjective perception of their personal worth and overall capabilities, and its formation is simultaneously affected by internal individual traits and external multiple environmental conditions; the internal level includes personal inherent characteristics such as personality traits and psychological endowments, while the external level covers multiple external cues such as hospital organization and management environment, recognition of professional value within the industry, and public social evaluation (25). Respect and affirmation from patients, colleagues, managers, and society help enhance nurses’ sense of self-worth. At the same time, a low-support, high-pressure, or low-recognition work environment may adversely affect their self-esteem (26).

In addition, the work engagement score was 27.27 ± 6.53 points, indicating a moderate level of vigor, dedication, and absorption at work. This result is consistent with existing studies (19). The dispersion also suggests differences in work enthusiasm and concentration among nurses. Some nurses can maintain high engagement, while others may show lower engagement due to work pressure, occupational burnout, limited development, or insufficient organizational support. Therefore, nursing managers need to address the different needs of nurse groups, rather than focusing only on the overall average level.

### 4.2 Effects of demographic characteristics on perceived decent work, self-esteem, and work engagement

After adjustment for age, household registration, education, and professional title, male nurses had lower perceived decent work (*B* = −3.447, 95% CI −5.606 to −1.288), self-esteem (*B* = −2.020, 95% CI −3.446 to −0.594), and work engagement (*B* = −2.656, 95% CI −4.524 to −0.789) scores than female nurses. These adjusted mean-score differences quantify the magnitude and uncertainty of the observed associations, but the small number of male participants (*n* = 59) limits precision and generalizability. Occupational gender stereotypes and role-identity conflict are plausible explanations reported in prior research (27, 28), although the present cross-sectional data did not measure or test those mechanisms.

The null age association in the adjusted model warrants cautious interpretation. Older age brackets were under-represented, limiting precision, and covariate adjustment may have over-controlled career-stage variables on the causal pathway from age to psychological outcomes, attenuating the total age effect. The significant unadjusted 20–25 versus 26–29 year differences further suggest age effects may be mediated rather than absent. Longitudinal studies with broader age sampling are needed to disentangle aging, cohort, and career-stage effects. Early-career adaptation demands described in previous studies (29–31) offer hypotheses for future longitudinal work, not explanations established by the present data. Training, mentorship, and career-support programs may therefore be reasonable candidates for prospective evaluation rather than interventions whose effectiveness can be inferred from this study.

Regarding professional title, nurses’ perceived decent work was significantly lower than that of nurse practitioners, which may be related to the fact that low-title nurses undertake more basic, repetitive, and executive work in clinical practice, with relatively low work autonomy and professional achievement. At the same time, low-title nurses may be in a relatively disadvantaged position in terms of salary and benefits, promotion opportunities, and professional recognition, which may affect their positive perception of decent work. Su et al. (32) and Patrician et al. (37) demonstrated that organizational justice influences young nurses’ turnover intention through the sequential mediation of organizational climate and emotional labor. When nurses with lower professional titles perceive distributive, procedural, and interactional justice, they tend to develop a more positive organizational atmosphere and experience less emotional exhaustion, thereby strengthening their intention to remain in the organization. Professional title promotion not only reflects improved professional ability but also the organization’s recognition of nurses’ professional value. Therefore, establishing a fair, transparent, and sustainable career development mechanism is crucial for improving the perceived decent work of low-title nurses.

The participating hospitals represented national-commission, provincial, municipal, and district/county administrative affiliations. However, hospital affiliation was not analyzed as a subgroup, and the hospitals and nurses were not probability sampled. The study therefore cannot compare administrative levels or determine whether the observed scores reflect a system-wide structural pattern. Such comparisons require larger samples with center-level data, prespecified multilevel analyses, and broader geographic coverage (33, 34, 38, 39).

### 4.3 Correlation among perceived decent work, self-esteem, and work engagement

The three core variables were positively correlated, with the largest coefficient between perceived decent work and work engagement (*r* = 0.6368), followed by self-esteem and work engagement (*r* = 0.4061) and perceived decent work and self-esteem (*r* = 0.3361). These coefficients indicate that higher scores tended to co-occur within the sample; they do not show that one construct affected or improved another. Self-Determination Theory provides one possible framework for future temporal testing of how respect, fairness, recognition, and development opportunities may be related to psychological resources and engagement (35, 40), but alternative directions and shared determinants remain possible.

### 4.4 Statistical indirect association through self-esteem

The cross-sectional mediation analysis yielded an indirect estimate of 0.071 (95% CI 0.029–0.121), representing 11.1% of the total association. In this manuscript, ‘indirect association’ and ‘partial mediation’ refer only to statistical decomposition of contemporaneously measured variables. The analysis does not demonstrate that perceived decent work preceded a change in self-esteem or that self-esteem subsequently changed work engagement (42).

Theoretically, perceived decent work summarizes nurses’ appraisals of the workplace environment, professional dignity, and organizational recognition (9). Prior theory suggests that respect, development support, and perceived fairness may be related to positive self-evaluation and professional confidence (17, 25, 36, 41). These mechanisms are plausible explanations for the observed covariance pattern, but the present study did not establish temporal sequence or within-person change. Longitudinal repeated measures are needed to test whether changes in work conditions precede changes in self-esteem and engagement.

The indirect component was modest (11.1%), while the direct statistical component accounted for 88.9% of the total estimate. These proportions should not be interpreted as causal pathways. They may reflect direct associations between workplace appraisals and engagement, the relative stability of self-esteem, measurement overlap, shared method variance, reverse directionality, or unmeasured organizational and individual factors. The cross-sectional design cannot distinguish among these explanations.

Accordingly, staffing, scheduling, reward fairness, career development, professional respect, organizational support, and psychological resources should be viewed as candidate targets for prospective organizational research. The present associations can inform intervention design and outcome selection, but they do not show that any specific organizational or individual intervention will increase work engagement.

## 5 Implications for nursing management

### 5.1 Organizational and nursing management implications

Perceived decent work was positively associated with work engagement, and the model identified a modest statistical indirect association through self-esteem. These findings can help nursing managers identify workplace and psychological domains for assessment, but the cross-sectional evidence does not demonstrate that changing those domains will cause higher engagement, retention, care quality, or patient safety.

First, optimize the organizational environment to enhance perceived decent work. Managers should rationally allocate human resources, optimize shift scheduling systems, and reduce workload overload; establish a fair and transparent performance and incentive mechanism so nurses’ labor efforts and professional contributions receive timely recognition; and enhance nurses’ participation in clinical decision-making and quality improvement to strengthen their sense of professional value.

Second, use subgroup findings to guide further assessment rather than deterministic targeting. The adjusted male–female differences were quantified with confidence intervals, whereas the age and professional-title comparisons were exploratory and lacked effect estimates. Managers may examine whether male, early-career, and lower-title nurses experience distinct barriers, while avoiding the assumption that demographic characteristics themselves cause disengagement.

Third, self-esteem may be included as one outcome in prospective evaluations of organizational support, positive feedback, peer support, or psychological empowerment. Because the observed indirect association was modest and cross-sectional, individual psychological programs should not substitute for evaluating staffing, workload, fairness, safety, and career systems.

Fourth, establish a continuous monitoring mechanism. Incorporate perceived decent work, work engagement, occupational burnout, and turnover intention into routine assessments, and regularly identify high-risk groups. Analyze the causes alongside factors such as workload, staffing, and night-shift frequency, and develop targeted improvement measures to shift management from passive turnover to active prevention.

Fifth, foster a respectful organizational culture. Strengthen the professional value of nursing through system and culture construction, and promote equal collaboration between doctors and nurses. Establish prevention, reporting, and support mechanisms for violence and disrespectful behavior to provide nurses with a safe, respectful, and supportive work environment.

In summary, the results generate two complementary sets of management hypotheses: organizational conditions such as staffing, incentives, career pathways, and culture, and psychological resources such as self-esteem and perceived support. Their effects on engagement, retention, quality, or patient outcomes should be tested prospectively rather than assumed from the present associations.

### 5.2 Potential health workforce policy relevance and research priorities

The present results should not be interpreted as direct evidence for national or subnational policy change. At most, they identify candidate decent-work domains for future health workforce measurement, including staffing adequacy, working time and rest, remuneration, professional development, participation in workplace decisions, protection from violence and harassment, and confidential mental health support. Whether these domains predict engagement, retention, or service continuity should be tested in representative, longitudinal studies with prespecified analyses across sex, career stage, professional title, hospital level, and geographic area.

Any organizational or policy pilot informed by these domains should include a prospective comparison, baseline and follow-up measurement, transparent reporting of effect sizes and confidence intervals, and outcomes beyond self-report, such as retention, sickness absence, vacancies, safety events, or service continuity. These are priorities for evaluation, not conclusions that the current study establishes policy effectiveness or readiness benefits.

## 6 Limitations and future research directions

First, the cross-sectional design cannot establish temporal ordering, causal effects, or causal mediation; reverse directionality and unmeasured confounding remain possible. Second, hospitals and nurses were recruited through a nonprobability, convenience process limited to five tertiary Grade-A hospitals in Sichuan Province. Distribution through nurse representatives and voluntary online participation may have introduced selection bias, the center-level recruitment denominator was not available, and the sample should not be considered representative of nurses in Sichuan Province or China. The small male subgroup (*n* = 59) further limits subgroup generalizability. Third, all core variables were collected by self-report in the same questionnaire, creating risks of social desirability and common method bias. Procedural safeguards and Harman’s single-factor result reduce neither risk to zero, and future studies should incorporate temporal separation, multiple informants, and objective organizational outcomes. Fourth, the regression analyses did not provide full residual and influence diagnostics in the available report, and the age and professional-title comparisons lacked absolute effect estimates and confidence intervals. These exploratory findings require replication with complete diagnostic reporting, effect sizes, and correction for multiple comparisons. Finally, longitudinal and intervention studies should examine alternative mediators, including psychological capital, professional identity, and perceived organizational support, and should test whether changes in workplace conditions precede changes in self-esteem and work engagement.

## 7 Conclusion

In this regional convenience sample of nurses from five tertiary Grade-A hospitals, perceived decent work was positively associated with work engagement. The cross-sectional model identified a modest statistical indirect association through self-esteem (0.071, 95% CI 0.029–0.121; 11.1% of the total estimate). Because the study used contemporaneous self-report data and nonprobability sampling, it cannot establish causal mediation, representativeness, intervention effects, or policy effectiveness. Future representative longitudinal studies and prospectively evaluated organizational pilots should report complete model diagnostics, effect sizes, and confidence intervals.

## Data Availability

The original contributions presented in the study are included in the article/supplementary material, further inquiries can be directed to the corresponding authors.
